# Reduced length of nodes of Ranvier and altered proteoglycan immunoreactivity in prefrontal white matter in major depressive disorder and chronically stressed rats

**DOI:** 10.1038/s41598-023-43627-4

**Published:** 2023-09-29

**Authors:** José Javier Miguel-Hidalgo, Erik Hearn, Mohadetheh Moulana, Khunsa Saleem, Austin Clark, Maggie Holmes, Kashish Wadhwa, Isabella Kelly, Craig Allen Stockmeier, Grazyna Rajkowska

**Affiliations:** Department of Psychiatry and Human Behavior, University of Mississippi Medical Center, 2500 N. State Street, Jackson, MS 39216 USA

**Keywords:** Depression, Neuroscience, Myelin biology and repair

## Abstract

Major depressive disorder (MDD) and chronic unpredictable stress (CUS) in animals feature comparable cellular and molecular disturbances that involve neurons and glial cells in gray and white matter (WM) in prefrontal brain areas. These same areas demonstrate disturbed connectivity with other brain regions in MDD and stress-related disorders. Functional connectivity ultimately depends on signal propagation along WM myelinated axons, and thus on the integrity of nodes of Ranvier (NRs) and their environment. Various glia-derived proteoglycans interact with NR axonal proteins to sustain NR function. It is unclear whether NR length and the content of associated proteoglycans is altered in prefrontal cortex (PFC) WM of human subjects with MDD and in experimentally stressed animals. The length of WM NRs in histological sections from the PFC of 10 controls and 10 MDD subjects, and from the PFC of control and CUS rats was measured. In addition, in WM of the same brain region, five proteoglycans, tenascin-R and NR protein neurofascin were immunostained or their levels measured with western blots. Analysis of covariance and t-tests were used for group comparisons. There was dramatic reduction of NR length in PFC WM in both MDD and CUS rats. Proteoglycan BRAL1 immunostaining was reduced at NRs and in overall WM of MDD subjects, as was versican in overall WM. Phosphacan immunostaining and levels were increased in both in MDD and CUS. Neurofascin immunostaining at NRs and in overall WM was significantly increased in MDD. Reduced length of NRs and increased phosphacan and neurocan in MDD and stressed animals suggest that morphological and proteoglycan changes at NRs in depression may be related to stress exposure and contribute to connectivity alterations. However, differences between MDD and CUS for some NR related markers may point to other mechanisms affecting the structure and function of NRs in MDD.

## Introduction

Stress is a major risk factor for the onset and maintenance of depressive episodes^1,2^. Both major depressive disorder (MDD) in humans and chronic stress in animal models^3^ are associated with symptoms of anhedonia and helplessness or anxiety-like behaviors. In addition, depression and experimentally-induced stress share comparable cellular and molecular disturbances that involve neurons and glial cells in gray and white matter, particularly in prefrontal brain areas^4–9^. These neurobiological alterations are concomitant with abnormal connectivity between the prefrontal cortex (PFC) and other cortical and subcortical regions detected with functional and structural neuroimaging approaches^10,11^. Disturbances of brain connectivity may be partly dependent on synaptic alterations that involve neurons and astrocytes in the gray matter^12^. However, the afferent and efferent connectivity of the prefrontal cortex critically relies on the propagation of signals along axons, many wrapped by a coat of oligodendrocyte-derived myelin that allows for fast, efficient signal conduction. This propagation along myelinated axons is ultimately dependent on the presence of nodes of Ranvier (NRs), which are short myelin-devoid axon segments rich in voltage-gated sodium channels (Navs) that critically sustain the faithful regeneration of axon potentials down the axon^13^. An array of extracellular matrix (ECM) proteins, including a variety of proteoglycans largely derived from glia, closely surrounds the NR, interacting between them and with axonal membrane proteins such as neurofascin, to facilitate NR stabilization and function^14–16^. Thus, connectivity disturbances in the PFC in depression and stress may be influenced by pathology of the perinodal ECM components that are released by the processes of astrocytes and other glial cells. A majority of NRs is decorated with the distal portions of astrocytes processes, while a smaller but still significant proportion receive glial processes from cells containing neuron-glial antigen 2 (NG2), a chondroitin sulfate proteoglycan^15^. These two cell types appear to contribute greatly to the expression of specific ECM proteoglycans such as phosphacan, neurocan, versican, brevican, BRAL1 and others that collaborate with cell adhesion molecules in axonal and oligodendrocyte membranes to organize the clustering of voltage-gated sodium channels and other channels at the NRs and paranodal regions^13,17,18^.

In a mouse model of chronic unpredictable stress (CUS), previous studies observed reduced length of NRs in the corpus callosum (CC) and abnormal morphology of processes in cultured oligodendrocyte^19^. In the human brain, the same researchers employed diffusion tensor imaging and observed reduced fractional anisotropy in the anterior genu of the CC of human subjects with MDD as compared to non-psychiatric controls, which was interpreted as reflecting microstructural disturbance of CC axon bundles in depression. It is not yet known whether in the prefrontal white matter (WM) underlying the gray matter of human subjects with MDD or in regions of WM other that the CC there is reduction of length of NRs comparable to that observed in the mouse CC after prolonged stress. It is also unclear whether proteoglycans, major protagonists in the stabilization of aggregation of voltage-gated sodium channels (Navs)^13,18,20^ and possibly in the regulation of the ionic environment of NRs^21^, are altered in MDD or as a consequence of repeated exposure to stress. Herein, we sought to determine whether there is reduced length of NRs in the WM subjacent to the PFC gray matter in MDD and in rats subjected to CUS. Furthermore, we used immunohistochemistry and western blot-based protein determinations to ascertain whether proteoglycans phosphacan, neurocan, brevican, versican and BRAL1, as well as Tenascin R and associated NR axonal membrane protein neurofascin, are altered in the overall WM neuropil and around individual NRs in the PFC WM in MDD and in rats subjected to CUS. The above-mentioned proteoglycans were specifically targeted because according to NR-focused studies they are the main components of the extracellular matrix around nodes of Ranvier^13,20,22,23^ along the myelinated CNS axons, and there is evidence about their involvement in the assembly and maintenance of CNS NRs^17,19,21,24^.

## Material and methods

### Rat brain sample collection

Twelve adult male rats of the Sprague–Dawley strain with weights between 200 and 250 g, were purchased from Charles River, Wilmington, MA, and acclimated for one week to our animal facilities. Then rats were randomly separated into two groups of six animals each. Rats in one group were individually subjected to CUS following the protocol detailed in previous publications^9,25^, while those in the control group were only handled for a short while and daily returned to their cages for the same 35-day period. At the end of the 35 days, novelty-suppressed feeding (NSF) was tested and scored for latency to feeding in all rats by researchers naïve to the treatment group as previously described^25^ to ensure behavioral effectiveness of the CUS procedure. Latency to feeding during the allotted time in the NSF procedure was dramatically increased in CUS-exposed rats (600 ± 0.00 s) as compared to controls (229.83 ± 48.87 s), t_10_ = 7.57, p < 0.0001, without reductions in the overall locomotor activity. Protocols were applied according to the regulations of the Institutional Animal Care and Use Committee and the National Institutes of Health.

At the end of behavioral testing animals were euthanized by decapitation and the brain immediately removed and split into the two cerebral hemispheres, and the frontal poles of these hemispheres were dissected and frozen in dry ice. The frontal pole from one hemisphere (from the frontal tip to the anterior edge of the optic chiasm) per animal was then sectioned coronally into 20 µm-thick sections with a cryostat microtome and the sections mounted on slides and stored frozen for subsequent fluorescence immunohistochemistry. The other frontal block was stored at – 80 °C and eventually was homogenized. The homogenate was centrifuged at 14,000*g* and the supernatant collected for western blotting.

### Human brain sample collection

Protocols for the inclusion of postmortem brain blocks from autopsies of deceased human subjects arriving to the Cuyahoga County Medical Examiners, for tissue collection, and for interviews were approved by the Institutional Review Boards of University Hospitals of Cleveland and University of Mississippi Medical Center. Informed consent was obtained from legally defined next-of-kin for tissue collection and for informant-based retrospective diagnostic interviews. Other details on collection of brain tissue, retrospective psychological diagnosis and body fluid determinations are described in our previous publications^26,27^.

At autopsy, blocks of prefrontal cortex containing the orbitofrontal cortex (OFC) were dissected, rapidly frozen, and stored at – 80 °C, and the postmortem interval (PMI; time between death and freezing of tissue) was noted. The brain tissue blocks were obtained from 11 non-psychiatric control subjects and 11 subjects diagnosed with MDD (Table 1). The subjects in both groups were of comparable ages at the time of death, sex and postmortem interval (time between death and freezing of autopsy samples). Tissue pH was determined for each subject from brain tissue frozen at the time of autopsy. OFC blocks containing Brodmann’s area 47 and the underlying white matter were frozen using isopentane cooled in dry ice and kept stored at – 80 °C until processing for immunohistochemistry. Sections at a thickness of 20 µm were obtained in a cryostat and stored for subsequent immunohistochemistry and detection of various proteins at NRs and the surrounding WM tissue.

**Table 1:** List of demographic characteristics for human subjects in the study.

Control	Sex	Age	PMI	pH	IHC/WB		
1	M	27	29	7.09	IHC and WB		
2	F	27	15	7.01	IHC and WB		
3	F	29	25	6.64	IHC		
4	F	38	11	6.46	IHC and WB		
5	M	41	28	7.14	IHC		
6	F	42	7.25	5.62	WB		
7	F	49	20	6.81	IHC and WB		
8	F	51	22	6.3	IHC		
9	F	65	26	6.175	WB		
10	M	67	24	6.96	WB		
11	M	68	24	6.625	IHC and WB		
12	M	70	29	6.81	IHC		
13	F	74	32	5.98	WB		
14	F	75	32	6.43	WB		
15	M	78	22	6.775	IHC		
16	F	80	21	6.78	IHC		
	6M/10F	55.06	22.95	6.60			

MDD	Sex	Age	PMI	pH	IHC/WB	Age of onset	Duration
1	M	19	24	6.82	WB	14	5
2	M	20	10	6.21	IHC	19	1
3	M	33	18	6.79	IHC and WB	29	4
4	F	34	27	6.27	IHC	14	20
5	F	40	25	6.32	IHC and WB	35	3
6	F	42	12	6.84	IHC	26	16
7	M	42	44	6.675	WB	42	0.6
8	F	44	29	6.71	IHC	32	12
9	F	50	23	6.83	IHC	46	35
10	M	62	22	6.06	WB	49	13
11	M	65	24	6.515	WB	65	0.1
12	M	74	25	6.67	IHC and WB	50	24
13	F	78	25	6.94	IHC and WB	63	15
14	M	78	26	6.74	IHC and WB	78	0.3
15	M	82	12	6.46	IHC and WB	20	62
	9M/6F	50.87	23.07	6.59			

List of demographic characteristics for human subjects in the non-psychiatric control group (CONTROL) and the group of subjects diagnosed with major depressive disorder (MDD) that were included for immunohistochemical and western blot based determinations in this study. Subjects are listed according to the age at the time of death, the rank number at left referring only to the order in the age-based list. PMI refers to the postmortem interval between time of death and brain autopsy and collection. Brain samples from the white matter were processed by immunohistochemistry (IHC) and/or western blotting (WB) as indicated, according to availability. Age of onset and duration of MDD (in years) were based on estimations by clinicians according to medical histories and reports from next-of-kin. At the bottom of each group there is a count of males and females in each group and averages of age, PMI and brain tissue pH.

Selection of human PFC tissue for western blots: For some of the subjects included in the immunohistochemistry study (5 in the non-psychiatric group, 4 in the MDD group) there was no extra frozen samples of WM for western blots. Nevertheless, punches of the ORB area 47 WM were still available for 6 additional non-psychiatric and 5 MDD subjects with ages, PMI and tissue pH that were comparable to those subjects used for immunohistochemistry, eventually allowing for the collection of tissue punches from area 47 WM of 10 control and 10 MDD subjects for western blot-based protein determinations (see Table 1 for the assignments of subjects to different experiments). Punches of tissue of about 80 mg each were made from the WM of cortical area 47, collected, homogenized and their supernatants obtained after centrifugation to determine the levels of proteoglycans and other proteins of interest. Lists of medical and demographic characteristics of the human subjects used for immunohistochemistry and western blots are presented in Table 1. Table 2 lists the primary antibodies used in the immunohistochemical and western blot determinations for human and rat samples, and their antigen specificities.

**Table 2:** List of primary antibodies used for detection and quantification of immunoreactivity for proteins included in the present study, arranged in alphabetical order of the antigen name, indicating whether the antibodies were used for rat or human samples and whether they were applied to samples processed for immunohistochemically or for western blots.

Molecular specificity	For detection of proteins in human, rat or both	IHC, WB or both	Species raised in/type	Dilution	Supplier	Cat. no
Anti-BRAL1	Both	Both	Rabbit/polyclonal	WB 1:5,000; IHC 1:1000	Abnova	PAB13027
Anti-brevican	Both	Both	Sheep/polyclonal	IHC 1:1,000; WB human 1:2000, rat 1:2000	R&D Systems	AF4009
Anti-CASPR	Both	IHC	Mouse/monoclonal	1:1000	Millipore	MABN69
Anti-Nav 1.6	Both	IHC	Rabbit/polyclonal	1:1000	Millipore	AB5580
Anti-neurofascin	Human	WB	Rabbit monoclonal	1:5,000	Abcam	ab184377
Anti-neurofascin	Both	Human IHC, rat both	Chicken/polyclonal	1:1,000	R&D Systems	AF3235
Anti-phosphacan	Human	Both	Rat/monoclonal	1:1,000	R&D Systems	MAB26881
Anti-phosphacan	Rat	Both	Mouse/monoclonal	1:2,000	Millipore	MAB5210
Anti-tenascin-R	Human	Human both; rat IHC	Goat/polyclonal	1:1,000	R&D Systems	AF3865
Anti-tenascin-R	Rat	WB	Mouse/monoclonal	1:500	R&D Systems	MAB1624
Anti-versican	Both	Both	Rabbit/monoclonal	1:500	Novus Biotech	NBP2-75706

The dilution of the antibody in the working solution, the supplier and the supplier catalog number at the time of purchase are also provided. BRAL1 is brain link protein 1, also known as HAPLN2 or hyaluronan and proteoglycan linkprotein 2; N_av_1.6 refers to the alpha subunit 1.6 of voltage-gated sodium channel; CASPR refers to contactin associated protein 1.

### Immunohistochemistry and morphometry

Sections from rat or human brain were mounted onto slides, and processed for immunofluorescence using primary antibodies to phosphacan, neurocan, brevican, versican, brain link protein 1 (BRAL1), Tenascin R, CASPR, neurofascin or Nav 1.6 (Table 2). Secondary antibodies (made against the IgG or IgM of the species of the primary antibody) conjugated with fluorochromes of different fluorescent emission spectra so as to label NRs were used throughout. Typically, an experiment with a particular histological section included three different primary antibodies: one against CASPR to label the paranodal regions enclosing the NRs, one against Nav 1.6 or neurofascin to identify the middle of the NR between the paranodal regions, and a third one to detect one of the proteoglycans or tenascin R.

Morphometry: Using a Leica SP8 super-resolution laser confocal microscope (Leica Biosystems, Deer Park, IL, USA) a total of 27 sampling sites in three different sections per subject were obtained by systematic random sampling in the WM under the medial aspect of PFC. Each sampling site was focused at 6 µm below the surface of the section under the microscope and a photomicrograph was collected. NRs were identified by pairs of CASPR-labeled paranodal regions enclosing a patch of Nav1.6 or neurofascin immunoreactivity. Within the micrograph the length of all identifiable NRs was measured as the distance between the edges of CASPR-positive paranodal regions encasing the Nav1.6 or neurofascin immunoreactive region.

In addition, sections with NRs immunofluorescently labeled for CASPR and Nav6 or neurofascin were labeled for one of proteoglycans (neurocan, phosphacan, brevican, versican, BRAL1), tenascin R or neurofascin. Micrographs from each combination of labeling (i.e., two markers for NRs, and proteoglycan or neurofascin) had all their NRs identified and a small rectangle was centered in the middle of each NR within the picture to determine the area average fraction of proteoglycan (illustrated in Figs. 2 and 3), neurofascin or tenascin R immunoreactivity contained within each rectangle in the micrograph. Rectangle dimensions were 4 × 2 µm for both human and rats with the major axis along the axon, with the exception of rectangles to measure neurofascin at the node, which was measured in a smaller box of 1.3 × 1.2 µm to exclude, as much as possible, neurofascin labeling at the paranodes. In addition, the overall area fraction of immunolabeling for a particular protein or proteoglycan in each of the same 27 micrographs per subject was also measured to have an estimate of the combined nodal and extranodal immunolabeling. To calculate the area fraction of immunoreactivity either in overall or in NR-centered rectangles, all the pictures corresponding to the immunofluorescence of a particular protein of interest for each subject in a particular experiment were taken by setting the same intensity of the selected incident laser light, and the same gain at the fluorescence detector. Pictures from the detector channel revealing the fluorescence of the protein of interest were compared to the pictures of the same fields containing immunofluorescent labeling of NRs for all the detector channels (three of four detector channels). The rectangle of interest (selection box) described above was drawn around each of the identified NR in the picture with all wavelength channels and the selection box transferred to the corresponding locations in the single channel detector picture containing only labeling for the protein of interest. All pictures with selections were then converted to grey level pictures with the ImageJ software (gray levels from 0 to 255), and, to identify specific immunoreactivity, the threshold above non-immunoreactive background was determined automatically using the maximum entropy algorithm available in ImageJ, to prevent subjective investigator decisions about background levels. Within each selection, the area of immunoreactivity (signal above threshold) was measured in ImageJ and divided into the total area of the selection box. The ratio of the area of fluorescent labeling within the selection to the total area of the selection was the area fraction of immunoreactivity around the NR, and was averaged for all the images in a particular picture and further averaged across pictures from a subject to provide the value of the variable used in statistical comparisons. Likewise, an area fraction was obtained for the immunoreactivity of a particular protein within the overall micrograph of brain tissue containing the NRs and averaged across images to provide the final value per subject. Thus, this overall immunoreactivity includes both the immunoreactivity around NRs and that beyond the selections around NRs). Note that laboratory staff were blind to the cohort of particular subjects.

### Western blotting and protein level determination

Western blotting: Rat frontal poles (approx. 90 mg) from the right cerebral hemisphere or punches (approx. 80 mg) from the WM under the human postmortem orbitofrontal cortex were homogenized for each subject. Individual homogenates were centrifuged at 14,000 rpm and the supernatants collected in homogenization buffer. Total protein in the supernatant was measured using the bicinchoninic acid assay and a plate reader set to measure absorbance at 570 nm. Samples were stored at – 80 °C. Between 1 and 30 µg of total protein per sample were loaded on individual wells of precast bis–Tris gels, and run by electrophoresis to separate protein bands. In each gel, half of the wells were loaded with samples from control subjects and the other half from subjects with MDD (or from rats exposed to stress), control samples alternating with human MDD diagnosis samples or with samples from CUS exposed rats. Gels were run in triplicate and blotted onto polyvinylidene difluoride (PVDF) membranes and these membranes were incubated with one of the primary antibodies to the target proteins of interest together with an antibody for a housekeeping protein. After washing, alkaline-phosphatase (AP)-conjugated secondary antibodies were applied and their binding revealed with substrates producing chemiluminescence in the presence of alkaline-phosphatase. Bands were detected and imaged in a Kodak Image Station 440 CF (Kodak, Rochester NY, USA). In each membrane, the binding of house-keeping proteins beta-actin or GAPDH was also revealed by chemiluminescence by adding specific antibodies, and used as a loading control. In preliminary experiments, no differences in optical density of actin or GAPDH were detected between control and MDD subjects, or between control and stressed rats. The relative levels of specific proteins were calculated by dividing the optical density of the bands for the proteins of interest into the optical density corresponding to the actin (or GAPDH) band. The final value for the level for a particular protein for a particular subject was the average of the triplicate values and used for subsequent statistical comparisons.

### Statistical analysis

Values of NR length, and area fraction of immunoreactivity for each of the proteins of interest were used as variables in statistical analyses. In rats, NR length and area fraction were compared between CUS exposed and non-exposed groups using a two-tailed Student’ t-test. Comparison of proteins levels determined with WB between controls and CUS-exposed rats were based on paired Students t-tests pairing the lane of a particular control rat with the adjacent lane of the CUS rats, to account for possible effects of position in the blot since lanes were arranged with alternating samples from control and CUS subjects. Differences between groups were considered significant at p < 0.05. Comparison of morphological variables or western blotting-based protein level data between human control and MDD subjects were analyzed using analysis of covariance whenever a correlation of potential covariates age, pH or PMI with the independent variable (NR length and area fraction) was found, or ANOVA if no correlation was detected. Possible correlation between variables was examined with Pearson correlation analysis and considered significant at p < 0.05.

### Ethics statements

Protocols for animal experiments and animal care were performed following the guidelines and regulations of the Institutional Animal Care and Use Committee and the National Institutes of Health and were approved by the Institutional Animal Care and Use Committee of the University of Mississippi Medical Center, Jackson, MS. The methods in animal studies were reported according to ARRIVE guidelines.

### Collection of human brain samples

Collection of postmortem human brain samples was performed according to protocols approved by the Institutional Review Boards of the University Hospitals of Cleveland, Ohio and the University of Mississippi Medical Center, Jackson, Mississippi and performed according to the approved guidelines and regulations of the National Institutes of Health. The protocol contained the requirement of obtaining written informed consent from next-of-kin for retrospective interviews to gather diagnostic information following methods described in detail in past publications^7,28^. Informed consent was obtained from all subjects and/or their legal guardian(s).

## Results

### Length of nodes of Ranvier in the PFC white matter

**Figure 1:**
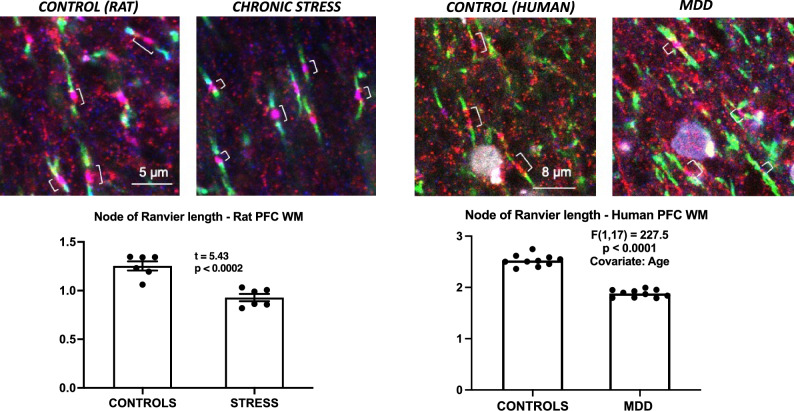
Representative micrographs, and charts illustrating the average length of nodes of Ranvier (brackets) in controls and CUS exposed rats (left two micrographs and bottom chart) and in control and MDD human subjects (right two micrographs and bottom chart) demonstrating significantly shorter nodes of Ranvier in animals with stress and human subjects with MDD.

The average length of NRs (distance between the two CASPR-labeled paranodes) (Fig. 1) was measured in the WM underlying the medial PFC anterior to the rostral tip of the genu of the corpus callosum in coronal sections of the rat brain of 6 rats exposed to CUS during 35 days and 6 control rats not exposed to CUS. NR length (Fig. 1) was significantly shorter by 26 percent in rats subjected to CUS as compared to control, non-CUS rats (Fig. 1). We also determined this length at a more rostral level of the rat PFC, where the sampled sites were mostly located in the interface between gray and white matter, and there was also a significantly shorter NR length in CUS rats as compared to controls.

In human brain samples, we imaged the WM from immunofluorescently labeled sections through the ventrolateral aspect of the OFC approximately at 200 µm from the WM/gray matter boundary. The NR length was measured in 11 subjects with MDD and 11 non-psychiatric (control) subjects. Remarkably, the average length in MDD subjects was found to be also about 26% shorter than in non-psychiatric controls.

### Area fraction of immunoreactivity and levels for proteoglycans and associated proteins at NRS rats exposed to CUS and human subjects with MDD

Using samples in sections immediately adjacent to where NR lengths were measured, we also determined the extent of immunoreactivity for proteins neurofascin and tenascin R, and for 5 proteoglycans (phosphacan, neurocan, versican, brevican and BRAL1) that have been described as components of the perinodal extracellular matrix^24^. The extent of immunoreactivity for proteins of interest as appearing in micrographs of fluorescently labeled white matter was determined using two different approaches: (1) by measuring the area fraction of the overall immunoreactivity for a particular protein in the whole sample (micrograph); and (2) by measuring the area fraction of immunoreactivity within a 4 × 2 µm rectangular window only around each individual node within a micrograph. Prefrontal white matter homogenate samples from MDD subjects and non-psychiatric controls and from frontal poles of CUS-exposed and control rats were also processed to determine levels of the same proteins using for the most part the same antibodies as in immunohistochemical studies (Table 2). The following paragraphs describe and report area fraction measurements of immunostaining and WB-based determinations of protein levels for the proteins of interest in rat and human brain tissue samples.

### Phosphacan

**Figure 2:**
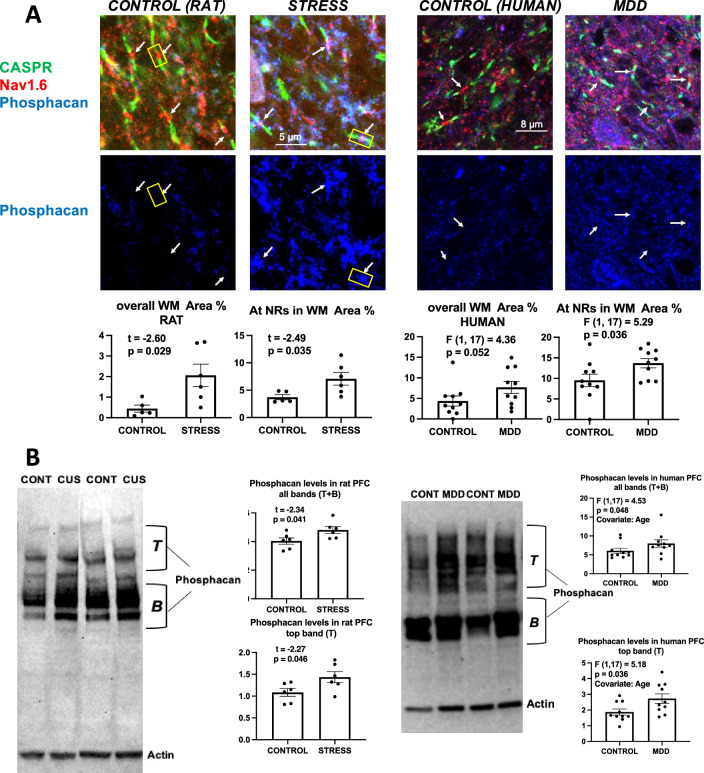
Phosphacan labeling in histological sections and western blots of human and rat prefrontal samples. (**A**) Micrographs and charts illustrating the average area fraction of immunoreactivity of phosphacan immunoreactivity (middle row of pictures) in controls and CUS exposed rats (left four micrographs and bottom charts) and in control and MDD human subjects (right for micrographs and bottom chart) demonstrating significantly higher immunoreactivity in overall and within 8 µm^2^ rectangles (“At NRs” graphs) around NRs in animals with stress and human subjects with MDD. Arrows point to nodes of Ranvier identified in the upper row of pictures using triple immunofluorescent staining for CASPR (labels paranodes in green) Nav1.6 (red) and phosphacan (blue in all the micrographs). Representative rectangles are shown drawn around nodes of Ranvier in the pictures on the left to eventually measure the immunoreactivity of the proteoglycan or protein with the picture containing only the label for the proteoglycan (blue in these images). Rectangles with the same dimensions were used around all NRs identified within all images of human and rat samples for all proteoglycans and proteins of interest in the present article. Calibration bar is 5 µm for rat micrographs and 8 µm for human micrographs. (**B**) Representative western blot lanes and quantification of optical density of phosphacan bands (relative to housekeeping protein actin) in the frontal pole of control and CUS rats and in the PFC white matter of human subjects. The pictures present top (T) and bottom (B) bands from 2 representative rats subjected to stress as compared to 2 controls (picture to the left) and two human MDD subjects against two non-psychiatric controls (picture to the right). Quantification of both T and B bands combined and for only the T band in all rats and in all humans in each group in the study are presented in the charts. All bands in the pictures appear as they were positioned and developed in the Western blot PVDF membrane. Note that in this and the remaining figures rat western blots are from blocks of tissue containing both gray and white matter, while human western blots were only from white matter tissue.

Overall area fraction of immunoreactivity for phosphacan was larger in CUS rats as compared to control rats (Fig. 2A). Likewise, the average area fraction around single NRs was significantly larger in CUS rats as compared to controls (Fig. 2A). In the human WM the area fraction around NRs was also significantly larger and there was a trend for overall fraction to be larger in subjects with MDD as compared to controls. (ANCOVA, PMI as covariate) (Fig. 2A charts).

**Figure 3:**
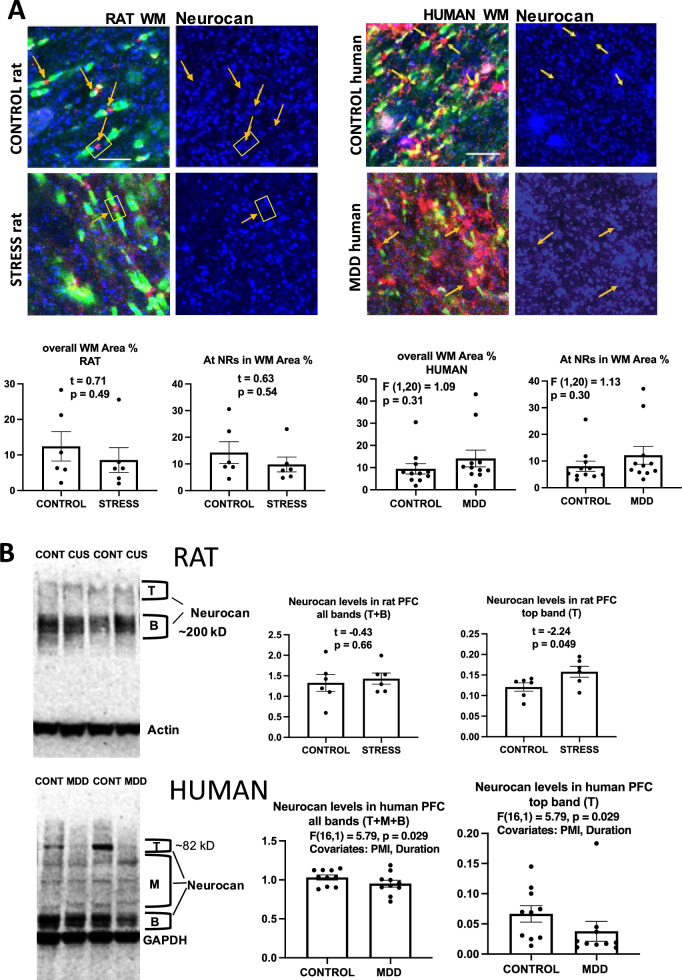
Neurocan labeling in histological sections and western blots of human and rat prefrontal samples. (**A**) Micrographs and charts illustrating the average area fraction of neurocan immunoreactivity in controls and CUS-exposed rats (four micrographs to the left and underlying charts) and in control and MDD human subjects (four micrographs to the right and underlying charts) showing no differences in overall Tenascin R immunoreactivity and within 8 µm^2^ rectangles (“At NRs” graphs) around individual NRs both in animals with stress and human subjects with MDD. Arrows point to the location of individual nodes of Ranvier identified in the left two pictures using triple immunofluorescent staining for CASPR (labels paranodes in green or yellow) Neurofascin (red) and Neurocan (blue in all the micrographs). Representative rectangles are shown drawn around nodes of Ranvier in the pictures on the left to eventually measure the immunoreactivity of the proteoglycan or protein with the picture containing only the label for the proteoglycan (blue in these images). Rectangles with the same dimensions were used around all NRs identified within all images of human and rat samples for all proteoglycans and proteins of interest in the present article. Calibration bar is 5 µm for rat and 8 µm for human micrographs. (**B**) Representative western blot lanes and quantification of optical density of Neurocan bands (relative to housekeeping protein actin for rat tissue or to GAPDH for human tissue) in the frontal pole of control and CUS rats and the PFC white matter of human subjects. The pictures present bands from 2 representative rats subjected to stress as compared to 2 controls (top picture; charts include all subjects in each group in the study) and from the prefrontal WM of two MDD subjects against two non-psychiatric controls (bottom picture and charts). Quantification for Neurocan from all bands combined as indicated top (T band), middle (M band), bottom (B band), or only for the T band is presented in the charts. All bands and lanes in the pictures appear as they were positioned and developed in the western blot PVDF membrane. Antibodies for actin or GAPDH were incubated jointly with Neurocan antibodies for simultaneous detection of protein bands after ensuring that there was no overlap of Neurocan bands and the housekeeping protein bands.

In western blots (Fig. 2B), both antibodies to rat and human samples revealed several bands between molecular weight markers of about 500 to 100 kD (rats) or between markers of 500 to 80 kD (human), the bands being similarly arranged in the blotted membrane for both species (Fig. 3). Quantification of phosphacan levels relative to actin for top bands (Fig. 2B, T) and for all the bands combined (Fig. 2B, T + B in top panels) showed that in rats subjected to CUS there was an elevated level of phosphacan as compared to controls (paired t-tests). Likewise, in the samples from human PFC WM phosphacan levels were also elevated for the top bands and for all bands combined (ANCOVA with age as covariate) (Fig. 2B, bottom panels).

### Neurocan

There was no significant difference in the area fraction of immunoreactivity for neurocan between CUS and control rats or between the MDD and non-psychiatric subjects in PFC histological sections (Fig. 3A), even if most of the highest values were found in subjects from the MDD group. Western blots (Fig. 3B) showed that the antibody used for rat samples (raised in mouse) labeled bands at about 400 and 120 kD while the antibody for human samples (raised in rabbit) labeled bands between 80 and 45 kD (Fig. 3B). In rats, the level assessed by combining all bands relative to housekeeping protein actin did not differ between the control and the CUS groups, although the highest, weaker band was significantly more intense in rats under CUS than in controls (Fig. 3B). By contrast, in humans, the immunoreactivity for both the ~ 82 kD band and all bands combined (relative to GAPDH immunolabeling, used in humans in place of actin due to the actin band’s proximity to one of the neurocan bands) was significantly reduced in MDD as compared to non-psychiatric subjects when using PMI and duration of MDD (in MDD subjects) as covariates (Fig. 3B).

### Versican

**Figure 4:**
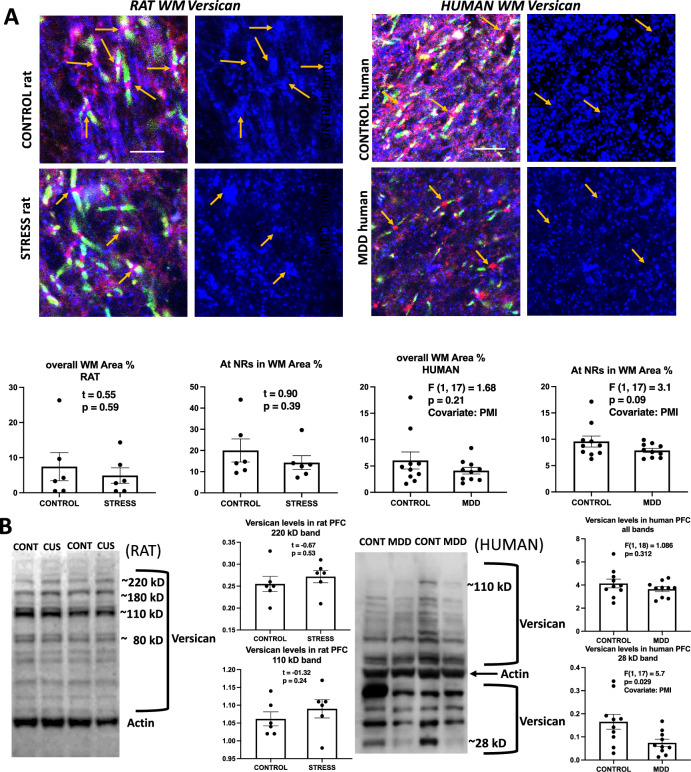
Versican labeling in histological sections and western blots of human and rat prefrontal samples. (**A**) Micrographs and charts illustrating the average area fraction of Versican immunoreactivity in control and CUS exposed rats (four micrographs to the left and underlying charts) and in control and MDD human subjects (four micrographs to the right and underlying charts) showing no differences in overall versican immunoreactivity and within 8 µm^2^ rectangles (see legends referring to rectangles in Figs. 2 and 3) around NRs both in animals with stress and in human subjects with MDD. Arrows point to nodes of Ranvier identified in the left two pictures using triple immunofluorescent staining for CASPR (labels paranodes in green) neurofascin (red) and versican (blue in all the micrographs). Calibration bar is 5 µm for rat and 8 µm for human micrographs. (**B**) Representative western blot lanes and quantification of optical density of versican bands (relative to housekeeping protein actin) in the frontal pole of control and CUS rats and in the PFC white matter of human subjects. The pictures present bands from 2 representative rats subjected to stress as compared to 2 controls (picture to the left) and two human MDD subjects against two non-psychiatric controls (picture to the right). Quantification for all bands combined and for specific bands at approximately 110 kD in rats and 28 kD in humans are presented in the charts. All lanes and bands in the pictures appear as they were positioned and developed in the western blot PVDF membrane.

A rabbit monoclonal antibody was used to detect immunoreactivity in histological sections of the PFC in both human subjects and experimental rats. In rats, area fraction of versican immunoreactivity was not significantly different in control as compared to CUS either in the overall WM neuropil or around NRs (Fig. 4A, top). In human subjects the same antibody revealed a similar pattern of staining. However, while the overall neuropil immunoreactivity was not significantly different in controls as compared to MDD subjects the immunoreactivity of versican around NRs was significantly smaller in MDD subjects as compared to non-psychiatric controls (Fig. 4A, bottom).

In PFC western blots (Fig. 4B) the rabbit antibody used labeled a multitude of bands above and below the band detected with the anti-actin antibody. None of the main bands was significantly different in immunostaining intensity when comparing controls to CUS rats (Fig. 4B). In humans, most bands in isolation or when grouped were not significanlty different between controls and MDD subjects, with the exception of the lowest band, which showed a significantly reduced level in MDD subjects as compared to controls (Fig. 4B; PMI as a covariate).

### Brevican

**Figure 5:**
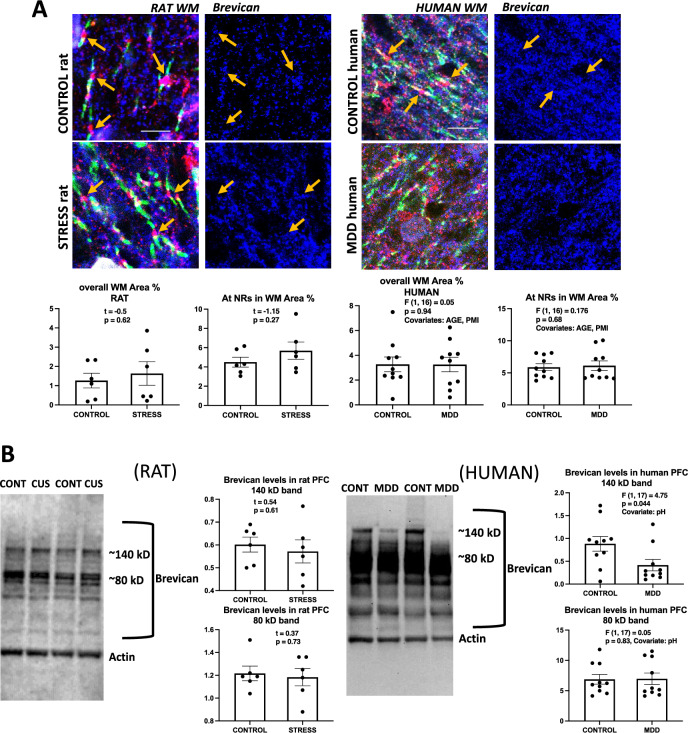
Brevican labeling in histological sections and western blots of human and rat prefrontal samples. (**A**) Micrographs and charts illustrating the average area fraction of brevican immunoreactivity in control and CUS exposed rats (four micrographs to the left and underlying charts) and in control and MDD human subjects (four micrographs to the right and underlying charts) showing no differences in overall brevican immunoreactivity and within 8 µm^2^ rectangles (see legends referring to rectangles in Figs. 2 And 3) around NRs both in animals with stress and in human subjects with MDD. Arrows point to nodes of Ranvier identified in the left two pictures using triple immunofluorescent staining for CASPR (labels paranodes in green) Neurofascin (red) and Brevican (blue in all the micrographs). Calibration bar is 5 µm for rat and 8 µm for human micrographs. (**B**) Representative western blot lanes and quantification of optical density of Brevican bands (relative to housekeeping protein actin) in the frontal pole of control and CUS rats, and in the PFC white matter of human subjects. The pictures present bands from 2 representative rats subjected to stress as compared to 2 controls (picture to the left) and two human MDD subjects against two non-psychiatric controls (picture to the right). Quantification for bands for all subjects in each group in the study at approximately 140 and 80 kD is presented in the charts. All lanes and bands in the pictures appear as they were positioned and developed in the Western blot PVDF membrane.

The area fraction of brevican immunoreactivity in the prefrontal WM was not statistically different between control and CUS rats or between control and MDD human subjects either in the overall WM or around NRs (Fig. 5A, top and bottom, respectively). In western blots (Fig. 5B), the same antibody (raised in sheep) used for immunohistochemistry was used to reveal brevican bands. In both human and rat western blots the antibody produced a comparable array of bands (Fig. 5B). In rats, there were no differences in the intensity of any of the defined bands relative to actin (Fig. 5B, left). In human brain, however, the band at ~ 140 kD was significantly less intense in MDD as compared to control subjects when including brain pH as a covariate, while there were no significant differences for the other bands (Fig. 5B, right).

### Bral1

**Figure 6:**
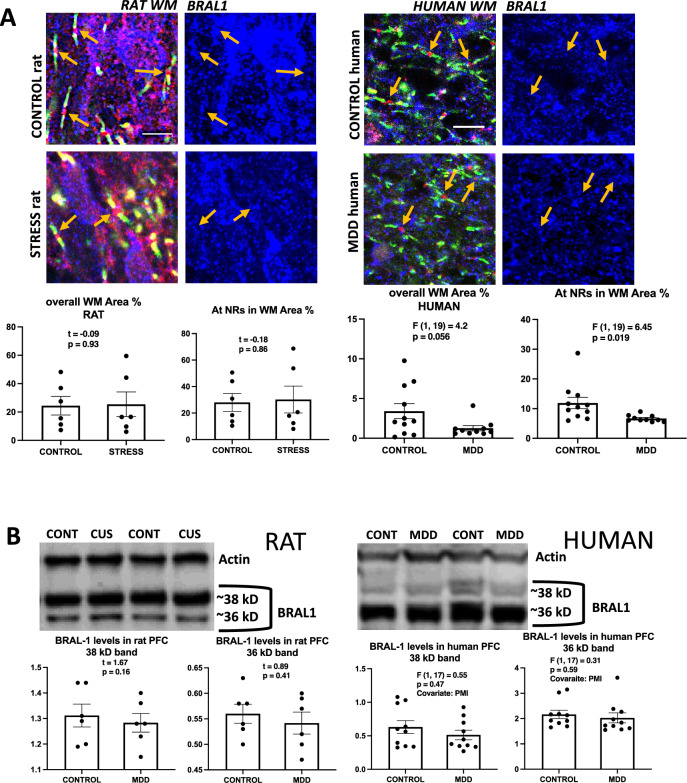
BRAL1 labeling in histological sections and western blots of human and rat prefrontal samples. (**A**) Representative micrographs and charts illustrating the average area fraction of BRAL1 immunoreactivity in rats (four micrographs to the left and underlying charts), and In human subjects (four micrographs to the right and underlying charts). There was significantly lower immunoreactivity within 8 µm^2^ rectangles (see legends referring to rectangles in Figs. 2 and 3) around NRs in MDD subjects as compared to non-psychiatric controls, and a tendency for lower fraction in the overall staining. No differences were observed between groups of rats. Arrows point to nodes of Ranvier identified in the three left pictures in the left using triple immunofluorescent staining for CASPR (labels paranodes in green), Neurofascin (red) and BRAL1 (blue in all pictures). Calibration bar is 5 µm for rat micrographs and 8 µm for human micrographs. (**B**) Representative western blot lanes and quantification of optical density of BRAL1 bands (relative to housekeeping protein actin) in the frontal pole of control and CUS rats and the PFC white matter of human subjects. The pictures present bands from 2 representative rats subjected to stress as compared to 2 controls (picture to the left) and two human MDD subjects against two non-psychiatric controls (picture to the right). Quantification of bands for all subjects in each group in the study at approximately 38 and 36 kD is presented in the charts. All bands in the pictures appear as they were positioned and developed in the Western blot PVDF membrane.

In sections of rat PFC, no significant differences were noted in the area fraction of Bral1 immunoreactivity in the overall WM and around NRs using an antibody made in rabbit (Fig. 6A, top). However, in human brain sections the same antibody exposed a significantly lower area fraction of Bral1 immunoreactivity both in the overall WM of the sections and around individual NRs in MDD vs. control subjects (Fig. 6A, bottom panel). With the same rabbit antibodies, western blots (Fig. 6B) from both human and rat samples revealed two bands at ~ 36 and ~ 38 kD, but in the rat the thickest band was ~ 36 kD while in human preparations the thickest band was at ~ 38 kD (Fig. 6). No significant difference was detected for either band between control and CUS rats (Fig. 6B, left). Likewise, no significant differences were observed in human western blots between control and MDD subjects for either band (Fig. 6B, right).

### Tenascin R

**Figure 7:**
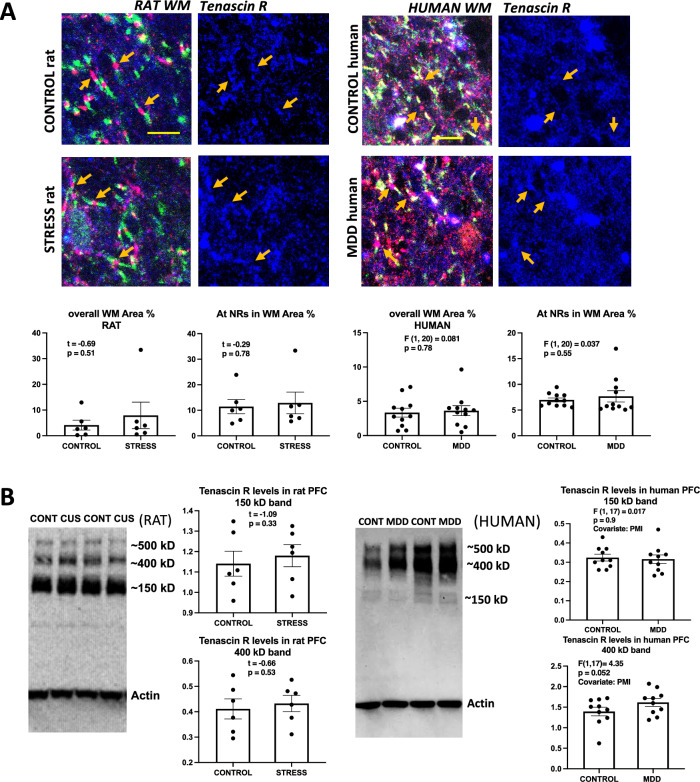
Tenascin R labeling in histological sections and western blots of human and rat prefrontal samples. (**A**) Representative micrographs and charts illustrating the average area fraction of Tenascin-R immunoreactivity in controls and CUS exposed rats (four micrographs to the left and underlying charts) and in control and MDD human subjects (four micrographs to the right and underlying charts) showing no differences in overall Tenascin R immunoreactivity and within 8 µm^2^ rectangles (see legends referring to rectangles in Figs. 2 and 3) around NRs both in animals with stress and human subjects with MDD. Arrows point to nodes of Ranvier identified in the left two pictures using triple immunofluorescent staining for CASPR (labels paranodes in green) Neurofascin (red) and Tenascin-R (blue in all the micrographs). Calibration bar is 5 µm for rat micrographs and 8 µm for human micrographs. (**B**) Representative western blot lanes and quantification of optical density of Tenascin R bands (relative to housekeeping protein actin) in the frontal pole of control and CUS rats and the PFC white matter of human subjects. The pictures present bands from 2 representative rats subjected to stress as compared to 2 controls (picture to the left) and two human MDD subjects against two non-psychiatric controls (picture to the right). Quantification for bands at approximately 150 and 400 kD is presented in the charts. All bands in the pictures appear as they were positioned and developed in the Western blot PVDF membrane. Note that rat samples are from blocks of tissue containing both gray and white matter, while human samples were only from white matter tissue.

To detect Tenascin R in rat PFC WM we used an antibody raised in mouse (see Table 2). This antibody revealed immunoreactivity throughout the WM, which was particularly enriched around NRs (Fig. 7A). However, no significant differences in area fraction of immunoreactivity between control and CUS rats were measured either in the overall picture or around NRs (Fig. 7A, top micrographs and charts). For human brain sections a goat-raised anti-Tenascin R antibody produced immunostaining in the WM neuropil similar to that in rats’ WM. Use of this antibody in human section did not reveal any significant difference between MDD and non-psychiatric subjects (Fig. 7A, bottom micrographs and charts).

In western blots (Fig. 7B), immunoreactivity for Tenascin R in rat PFC was probed with a goat polyclonal antibody. The bands were detected mainly between estimated molecular weights of about 120 kD and 500 kD, comprising a large band at about 200 kD and well-defined bands at approximately 400 and 500 kD (Fig. 7B). Levels relative to actin at those bands were not significantly different between control and CUS animals (Fig. 7B, left). In human PFC WM samples, the same antibody used for rats samples produced immunostaining at comparable molecular weight bands, where there was a trend for increased intensity for the 400 kD band in MDD subjects vs. controls (Fig. 7B, right).

### Neurofascin

**Figure 8:**
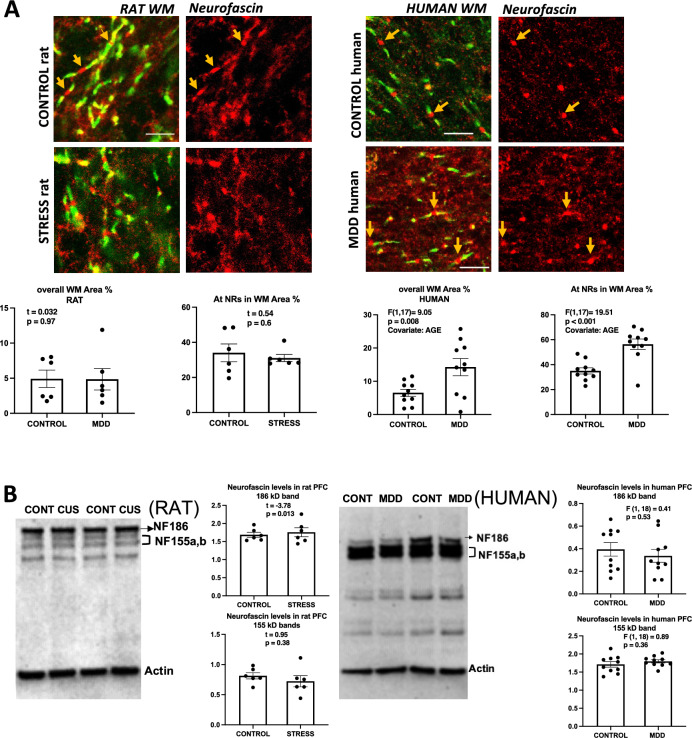
Neurofascin labeling in histological sections and western blots of human and rat prefrontal samples. (**A**) Micrographs and charts illustrating the average area fraction of Neurofascin immunoreactivity in rats (four micrographs to the left and underlying charts) and in MDD human subjects (four micrographs to the right and underlying charts) demonstrating significantly higher immunoreactivity in overall and within 4 µm^2^ rectangles (see legends referring to rectangles in Figs. 2 and 3) around NRs in human subjects with MDD as compared to non-psychiatric controls when using age at death as covariate. Arrows point to nodes of Ranvier identified in the three left pictures in the left using double immunofluorescent staining for CASPR (labels paranodes in green) and Neurofascin (red). Neurofascin is known to be heavily concentrated at nodes of Ranvier as axonal NF186 and in paranodes as oligodendrocyte-derived NF155). Calibration bar is 5 µm for rat micrographs and 8 µm for human micrographs. (**B**) Representative western blot lanes and quantification of optical density of Neurofascin bands (relative to housekeeping protein actin) in the frontal pole of control and CUS rats (left) and the PFC white matter of human subjects right. The pictures present bands from 2 representative rats subjected to stress as compared to 2 controls (picture to the left) and two human MDD subjects against two non-psychiatric controls (picture to the right). Quantification for bands at approximately 186 and 155 kD is presented in the charts. All bands in the pictures appear as they were positioned and developed in the Western blot PVDF membrane. NF186 denotes the band of the higher molecular form of neurofascin, most likely of neuronal/axonal origin and NF155 denotes bands of lower molecular weight most likely corresponding to paranodal neurofascin, which is mostly generated by oligodendrocytes. Note that rat samples are from blocks of tissue containing both gray and white matter, while human samples were only from white matter tissue.

Determination of neurofascin (NF) area fraction took into account that the isoform NF186 is a neuronal membrane-spanning protein heavily concentrated at the node of Ranvier, while the NF155 form is expressed by oligodendrocytes at high levels in the oligodendrocyte membranes at paranodal and juxtaparanodal portions of myelin^16^. Since the antibody we used labels NF in both nodal (NF186) and extranodal (NF155) regions^18^, the nodal immunoreactivity was first determined by drawing a rectangular selection smaller than in determinations of the other proteins and that in most cases contained only the length of the node, and excluded the paranodes. The area fraction of NF immunoreactivity around each of the individual nodes was computed and averaged for each sample. This nodal NF fraction was not significantly elevated in the stressed rats as compared to their controls (Fig. 8A, top), but was significantly increased in MDD as compared to control subjects (Fig. 8A, bottom), respectively. Area fraction of overall NF immunoreactivity (thus would include all nodal, paranodal and extranodal immunoreactivity) was significantly increased in humans with MDD there as compared to their non-psychiatric controls (Fig. 8A, “overall” charts).

In western blots (Fig. 8B) of the human PFC WM and from the PFC of rats the antibody used also detected a top band likely corresponding to about 186 kD and two close bands likely representing 155 and 140 kD bands (Fig. 8B), The top band was the most intense in rats (Fig. 8B, left), while in humans the other two bands displayed higher density than the top band (Fig. 8B, right). Immunoreactivity level of the top band (186 kD) was significantly higher in CUS rats as compared to controls (Fig. 8B, left). In human samples, no significant differences in the intensity of the top band or the other bands was measured when comparing MDD to non-psychiatric control subjects (Fig. 8B, right).

## Discussion

The present report provides morphometric and western blot-based evidence that the in situ immunoreactivity and the estimated levels of some ECM proteoglycans and axonal protein NF186 are significantly altered in CUS and MDD either as a consequence of stress or due to pathological processes underlying MDD. The altered levels and/or distribution may have an impact on the structure and function of individual NRs because altered immunoreactivity of phosphacan and NF186 within a small window (8 µm^2^) around individual nodes of Ranvier was detected after CUS in rats, and the same molecules were also altered around individual NRs in the brains of human subjects with MDD diagnosis. Moreover, some of the western blot data were consistent with the direction of changes detected with immunohistochemistry suggesting that alterations in the turnover of the studied proteins is reflected in their distribution around NRs. In addition, the present study also revealed a decrease of the average NR length in the PFC white matter in CUS-exposed rats and in humans with MDD. This is consistent with previous reports that NR length was reduced in the corpus callosum of CUS mice^19^, and further suggests that stress-related mechanisms leading to structural adaptations at NR may also participate in neuropathological changes at NRs in the prefrontal WM in MDD. In addition, our data suggest that in vivo neuroimaging-based differences detected in axon bundle structure as assessed by diffusion tensor imaging in the CC in MDD^19^, may be partly related to NRs length or other NRs structural and molecular changes in the orbitofrontal white matter in postmortem tissue from subjects with MDD.

Stress is a major risk factor for MDD episodes, and in animal models the behavioral consequences of prolonged stress such as anhedonia (failure to seek normally reinforcing stimuli), helplessness (failure to act against or escape from threats) or anxiety-like behavior appear to present some face value in modeling similar behaviors in humans^4,29–31^. There are also commonalities in the morphological and neurochemical alterations of glial cells in prefrontal cortical areas in CUS and MDD, such as reduced GFAP or connexin 43 immunoreactivity or decrease in the expression of some myelin related proteins^5,7,22,32^. If these behavioral and neurobiological similarities between CUS and MDD depend on shared pathophysiological mechanisms, it is possible that stress-related NRs pathology contributes to the onset or maintenance of depression. It is also possible, though, that the etiology of depression or related symptoms depends initially on neuronal pathology, but that astrocytes and other glial cells respond to that pathology by altering the environment of NRs, thus contributing to the persistence of disturbed function that leads to the behavioral anomalies.

The length of NRs is likely to depend on the adhesion molecules at the axonal node membrane and the paranode regions on both sides of the NR, as well as on their interactions with the complex ECM around nodes^13,33^. Given that postmortem NRs length estimation may be biased by tissue collection procedures and treatments, determining with precision how the length may affect signal conduction and integration may be challenging, but general principles are starting to emerge from studies in animal models regarding the impact of myelin alterations and NR length on propagation and synchronization of signals^34,35^. Some of these studies of nodal ECM and axonal morphology, together with predictions based on theoretical approaches have found that optimal function of NRs in signal propagation and integration may depend on an adequate range of lengths, such that shorter or longer NRs may lead to dysfunctional integration of signals between connected brain centers^34^. Shortening of NRs may indicate diminished or aberrant plasticity of the paranodal myelin wrapping around axons or suboptimal arrangement of perinodal ECM and NR axonal components. For example, reduced plasticity may be promoted by excessive neurofascin-dependent enhancement of membrane attachments. Likewise, prolonged neurocan or phosphacan excess at the nodal ECM, which has been also found in models of traumatic brain injury and degeneration^23,36^, may interfere with normal function, although, clearly, the possibility that increases may be of a compensatory or protective nature cannot be ruled out. The time-dependent complexity of level changes for various proteoglycans detected in lesions of the spinal cord during the post-trauma period may also apply to protracted stress or psychopathological processes^23,36,37^. As the present study is only based on a cross-sectional (vs. longitudinal) sampling of brain structures, the importance of protracted WM proteoglycan and ECM changes and the exact role they play in the pathophysiology of depression and stress-induced brain dysfunction remains to be firmly established.

Despite comparable tendencies in MDD subjects and CUS rats for increased phosphacan and neurofascin detection against their respective controls, there was largely unchanged in situ immunolabeling for neurocan, brevican and versican, and lower Bral1 immunoreactivity in overall and at NRs, while in western blots signal from some neurocan and versican bands were lower in MDD as compared to non-psychiatric human subjects. By contrast, in CUS rats some of those differences appeared to be absent as compared to their controls. Consistency of some changes between MDD and CUS-related pathology may indicate that an active MDD diagnosis may cause or involve stress-related mechanisms that are partly reflected in specific axonal and ECM neuropathology and altered expression of some proteins at NRs. On the other hand, the differences between MDD and CUS effects suggest there is MDD-specific pathology in WM and NRs that could involve ECM proteoglycans in mechanisms not necessarily related to the stress response. Clearly, although stress is an important risk factor for depression, the onset and progression of depression in humans are related to a panoply of factors other than stress that may find their reflection in reduced expression or abnormal distribution of proteoglycans around NRs. In comparison to the vast and expected variety in disease trajectories in MDD-diagnosed subjects, it is also possible that the relatively homogeneous environments and short span of stress and post-stress periods in the experimental animals result in expected differences in pathophysiology between stress effects in rat models and the consequences of stress and other factors in MDD. Longitudinal changes in proteoglycan and associated axonal proteins were not directly addressed in the present research.

The apparent increase in phosphacan levels stands in contrast with the rather unchanged or slightly decreased levels of other examined proteoglycans. That contrast may be related to the specific functions and binding affinities of phosphacan as compared to the other proteoglycans^38^. In other models that have specifically targeted changes in several proteoglycans after spinal cord injuries the direction of changes for phosphacan is indeed opposite to changes in brevican, versican and neurocan^23^. In addition, longitudinal determinations of those changed proteoglycans demonstrated a time-dependency of alterations, suggesting that they are involved in an adaptive process. In lesions to the cerebral cortex, phosphacan and neurocan were found increased around glial scars but brevican and versican were not increased^36,37^. It remains to be ascertained if proteoglycan disturbances around NRs in stress or depression follow similar mechanisms or principles as observed in CNS injury. While phosphacan interacts with Tenascin R and Contactin-1^39,40^, brevican and versican rather bind hyaluronan through the linker protein BRAL1^41^. Tenascin R also binds NF186 and contactin-1 at the NRs, which in turn participate in the perinodal environment by linking to various components of the perinodal ECM^33,42^. Thus, one possible explanation for contrasting directions of change is that deficits in the stability of brevican, versican, neurocan or BRAL1 complexes bring about a compensatory phosphacan increase to offset reduced stability of ECM interactions around nodes. Lack of stability may influence maintenance of ion channel aggregation and localization at NRs or affect the ability to contain and restrain ionic movements essential to the adequate regeneration of action potentials^43^. Clearly, the exact nature and relevance of mechanisms responsible for differential proteoglycan responses in affective disorders will require further research.

Potential limitations and need for further studies: The present study involved numbers of animals and human subjects that although sufficient to reveal some significant differences may have been insufficient to fully assess the changes and involvement of particular mechanisms that may operate at NRs, and, thus, future studies with approaches allowing for higher throughput within a feasible time frame are needed. Further, immunohistochemical determinations in brain sections of rat and human subjects, and western blots from human tissue, were restricted to the white matter, while western blots from rat frontal poles contained both white and gray matter due to the limitations of the sampling procedure. Accordingly, while some of the findings in the present study are highly consistent between rats and humans and may be attributed to stress or MDD-related effects in the WM, comparisons to western blot-based levels of proteins of interest in the rat PFC may be affected by the influence of gray matter proteins and their changes in the rat PFC samples. Likewise, the direction of some changes in WB-based protein levels, as in the case of neurofascin, are apparently not consistent with the changes observed when measuring fraction of immunoreactivity in human subjects. This apparent discrepancy maybe partly due to the fact that, as indicated above, the antibody to neurofascin used does recognize various isoforms. It is also possible that there is a stress or depression related change in the axonal and glial distribution of neurofascin that, without affecting overall levels, results in alterations of the area fraction of immunoreactivity at NRs and beyond, since this morphological measurement only partially depends on protein level and is heavily influenced by distribution and morphology of cellular components within the tissue. At any rate, future studies using single-cell based determinations and more specific antibodies or other probes may allow for greater topological precision in determining PFC protein expression in the white matter underlying the prefrontal cortex in rats, and determining the fine distribution of immunoreactivity for the various proteins in neurons and glia (Supplementary Information).

### Supplementary Information

Supplementary Information.

## Data Availability

The datasets used and/or analyzed during the current study are available from the corresponding author on reasonable request.

## References

[1] Lupien SJ, McEwen BS, Gunnar MR, Heim C (2009). Effects of stress throughout the lifespan on the brain, behaviour and cognition. Nat. Rev. Neurosci..

[2] Swaab DF, Bao AM, Lucassen PJ (2005). The stress system in the human brain in depression and neurodegeneration. Ageing Res. Rev..

[3] Willner P (2017). The chronic mild stress (CMS) model of depression: History, evaluation and usage. Neurobiol. Stress..

[4] Slattery DA, Cryan JF (2017). Modelling depression in animals: At the interface of reward and stress pathways. Psychopharmacology..

[5] Rajkowska G, Miguel-Hidalgo JJ (2007). Gliogenesis and glial pathology in depression. CNS Neurol. Disord. Drug Targets..

[6] Miguel-Hidalgo JJ, Waltzer R, Whittom AA, Austin MC, Rajkowska G, Stockmeier CA (2010). Glial and glutamatergic markers in depression, alcoholism, and their comorbidity. J. Affect. Disord..

[7] Miguel-Hidalgo JJ, Wilson BA, Hussain S, Meshram A, Rajkowska G, Stockmeier CA (2014). Reduced connexin 43 immunolabeling in the orbitofrontal cortex in alcohol dependence and depression. J. Psychiatr. Res..

[8] Nagy C, Suderman M, Yang J, Szyf M, Mechawar N, Ernst C (2015). Astrocytic abnormalities and global DNA methylation patterns in depression and suicide. Mol. Psychiatry..

[9] Rajkowska G, Legutko B, Moulana M, Syed M, Romero DG, Stockmeier CA (2018). Astrocyte pathology in the ventral prefrontal white matter in depression. J. Psychiatr. Res..

[10] Zheng C, Quan M, Zhang T (2012). Decreased thalamo-cortical connectivity by alteration of neural information flow in theta oscillation in depression-model rats. J. Comput. Neurosci..

[11] Frodl T, Bokde AL, Scheuerecker J, Lisiecka D, Schoepf V, Hampel H (2010). Functional connectivity bias of the orbitofrontal cortex in drug-free patients with major depression. Biol. Psychiatry..

[12] Csabai D, Wiborg O, Czeh B (2018). Reduced synapse and axon numbers in the prefrontal cortex of rats subjected to a chronic stress model for depression. Front. Cell Neurosci..

[13] Rasband MN, Peles E (2015). The nodes of Ranvier: Molecular assembly and maintenance. Cold Spring Harb. Perspect. Biol..

[14] Arancibia-Carcamo IL, Attwell D (2014). The node of Ranvier in CNS pathology. Acta Neuropathol..

[15] Serwanski DR, Jukkola P, Nishiyama A (2017). Heterogeneity of astrocyte and NG2 cell insertion at the node of ranvier. J. Comp. Neurol..

[16] Susuki K, Chang KJ, Zollinger DR, Liu Y, Ogawa Y, Eshed-Eisenbach Y (2013). Three mechanisms assemble central nervous system nodes of Ranvier. Neuron..

[17] Melendez-Vasquez C, Carey DJ, Zanazzi G, Reizes O, Maurel P, Salzer JL (2005). Differential expression of proteoglycans at central and peripheral nodes of Ranvier. Glia..

[18] Amor V, Zhang C, Vainshtein A, Zhang A, Zollinger DR, Eshed-Eisenbach Y (2017). The paranodal cytoskeleton clusters Na^+^ channels at nodes of Ranvier. Elife..

[19] Miyata S, Taniguchi M, Koyama Y, Shimizu S, Tanaka T, Yasuno F (2016). Association between chronic stress-induced structural abnormalities in Ranvier nodes and reduced oligodendrocyte activity in major depression. Sci. Rep..

[20] Brivio V, Faivre-Sarrailh C, Peles E, Sherman DL, Brophy PJ (2017). Assembly of CNS nodes of ranvier in myelinated nerves is promoted by the axon cytoskeleton. Curr. Biol..

[21] Bekku Y, Oohashi T, Sango K, Yamauchi J, Ogata T, Susuki K (2019). Under the ECM dome: The physiological role of the perinodal extracellular matrix as an ion diffusion barrier. Myelin: Basic and Clinical Advances.

[22] Miguel-Hidalgo JJ, Moulana M, Deloach PH, Rajkowska G (2018). Chronic unpredictable stress reduces immunostaining for connexins 43 and 30 and myelin basic protein in the rat prelimbic and orbitofrontal cortices. Chronic Stress..

[23] Jones LL, Margolis RU, Tuszynski MH (2003). The chondroitin sulfate proteoglycans neurocan, brevican, phosphacan, and versican are differentially regulated following spinal cord injury. Exp. Neurol..

[24] Lubetzki C, Sol-Foulon N, Desmazières A (2020). Nodes of Ranvier during development and repair in the CNS. Nat. Rev. Neurol..

[25] Riaz MS, Bohlen MO, Gunter BW, Henry Q, Stockmeier CA, Paul IA (2015). Attenuation of social interaction-associated ultrasonic vocalizations and spatial working memory performance in rats exposed to chronic unpredictable stress. Physiol. Behav..

[26] Zhu H, Urban DJ, Blashka J, McPheeters MT, Kroeze WK, Mieczkowski P (2012). Quantitative analysis of focused a-to-I RNA editing sites by ultra-high-throughput sequencing in psychiatric disorders. PLoS ONE..

[27] Miguel-Hidalgo JJ, Overholser JC, Jurjus GJ, Meltzer HY, Dieter L, Konick L (2011). Vascular and extravascular immunoreactivity for intercellular adhesion molecule 1 in the orbitofrontal cortex of subjects with major depression: Age-dependent changes. J. Affect. Disord..

[28] Whittom A, Villarreal A, Soni M, Owusu-Duku B, Meshram A, Rajkowskai G (2014). Markers of apoptosis induction and proliferation in the orbitofrontal cortex in alcohol dependence. Alcohol. Clin Exp Res..

[29] Murphy-Royal C, Gordon GR, Bains JS (2019). Stress-induced structural and functional modifications of astrocytes—Further implicating glia in the central response to stress. Glia..

[30] Cathomas F, Azzinnari D, Bergamini G, Sigrist H, Buerge M, Hoop V (2019). Oligodendrocyte gene expression is reduced by and influences effects of chronic social stress in mice. Genes Brain Behav..

[31] Gould TD, Georgiou P, Brenner LA, Brundin L, Can A, Courtet P (2017). Animal models to improve our understanding and treatment of suicidal behavior. Transl. Psychiatry..

[32] Sun JD, Liu Y, Yuan YH, Li J, Chen NH (2012). Gap junction dysfunction in the prefrontal cortex induces depressive-like behaviors in rats. Neuropsychopharmacology..

[33] Rasband MN, Peles E (2021). Mechanisms of node of Ranvier assembly. Nat. Rev. Neurosci..

[34] Arancibia-Carcamo IL, Ford MC, Cossell L, Ishida K, Tohyama K, Attwell D (2017). Node of Ranvier length as a potential regulator of myelinated axon conduction speed. Elife..

[35] Ford MC, Alexandrova O, Cossell L, Stange-Marten A, Sinclair J, Kopp-Scheinpflug C (2015). Tuning of Ranvier node and internode properties in myelinated axons to adjust action potential timing. Nat. Commun..

[36] McKeon RJ, Jurynec MJ, Buck CR (1999). The chondroitin sulfate proteoglycans neurocan and phosphacan are expressed by reactive astrocytes in the chronic CNS glial scar. J. Neurosci..

[37] Tang X, Davies JE, Davies SJ (2003). Changes in distribution, cell associations, and protein expression levels of NG2, neurocan, phosphacan, brevican, versican V2, and tenascin-C during acute to chronic maturation of spinal cord scar tissue. J. Neurosci. Res..

[38] Faivre-Sarrailh C, Devaux JJ (2013). Neuro-glial interactions at the nodes of Ranvier: Implication in health and diseases. Front. Cell Neurosci..

[39] Falk J, Bonnon C, Girault J-A, Faivre-Sarrailh C (2002). F3/contactin, a neuronal cell adhesion molecule implicated in axogenesis and myelination. Biol. Cell..

[40] Fawcett JW, Oohashi T, Pizzorusso T (2019). The roles of perineuronal nets and the perinodal extracellular matrix in neuronal function. Nat. Rev. Neurosci..

[41] Cicanic M, Sykova E, Vargova L (2012). Bral1: "Superglue" for the extracellular matrix in the brain white matter. Int. J. Biochem. Cell Biol..

[42] Labasque M, Faivre-Sarrailh C (2010). GPI-anchored proteins at the node of Ranvier. FEBS Lett..

[43] Bekku Y, Vargova L, Goto Y, Vorisek I, Dmytrenko L, Narasaki M (2010). Bral1: Its role in diffusion barrier formation and conduction velocity in the CNS. J. Neurosci..

