# Supplementary material for: Reduced length of nodes of Ranvier and altered proteoglycan immunoreactivity in prefrontal white matter in major depressive disorder and chronically stressed rats
Source: Sci Rep. 2023 Sep 29;13:16419. doi: 10.1038/s41598-023-43627-4 (PMC10541441; doi:10.1038/s41598-023-43627-4)

Asterisks on top of blots mark the lanes used in the western blot illustrations of the figures, and red rectangles contain the actual parts of the original western blots used in the figures

FIG 2B

Phosphacan western blots

RAT

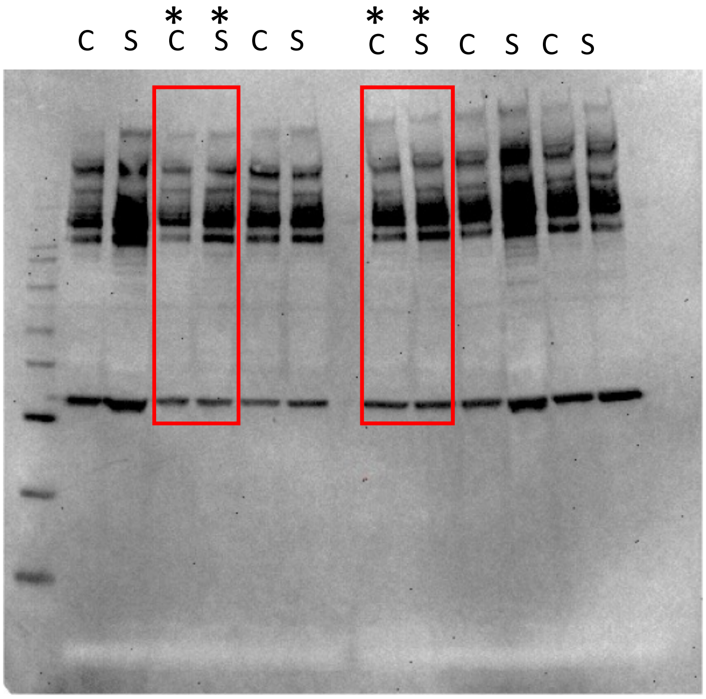

C=control
S=CUS

HUMAN

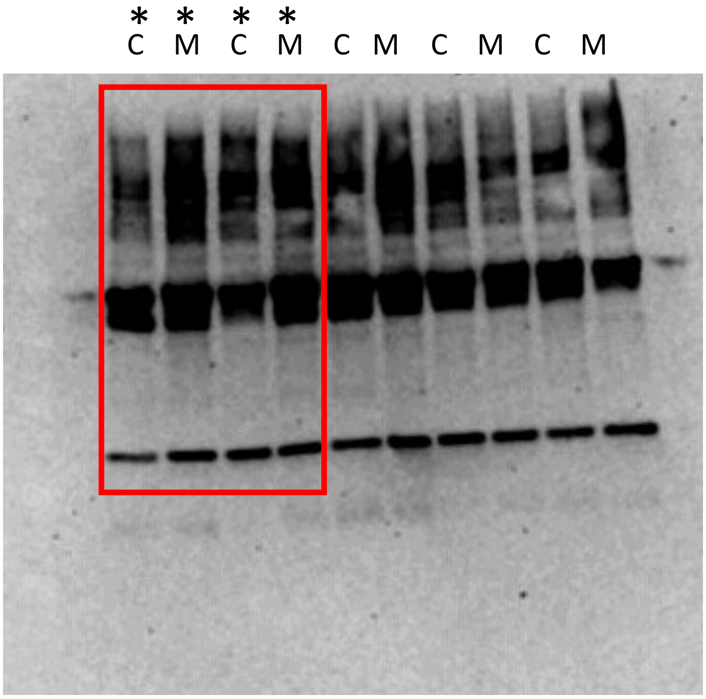

C=control
M=MDD

FIG 3B Neurocan western blots

RAT

* * * *
S C S C S C S C S C S C

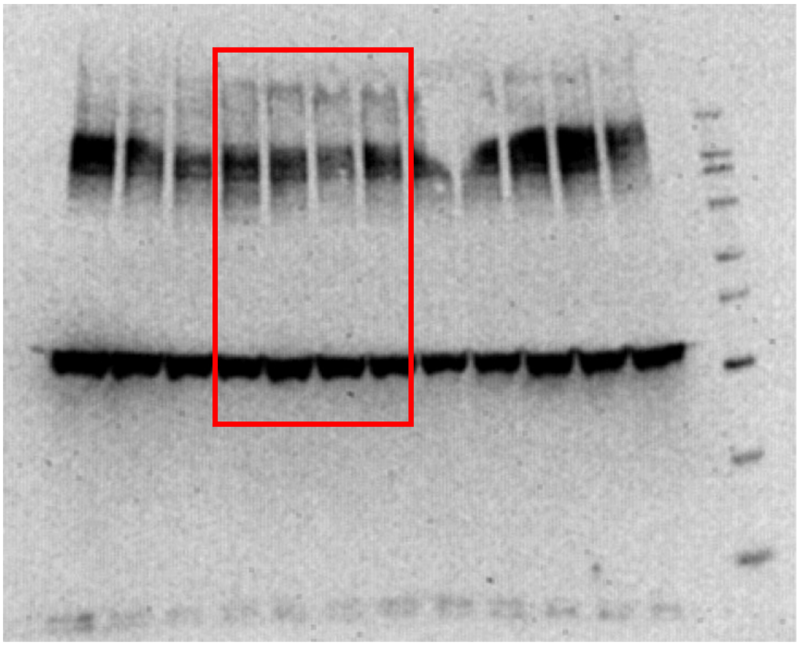

C=control
S=CUS

HUMAN

* * * *
C M C M C M C M C M

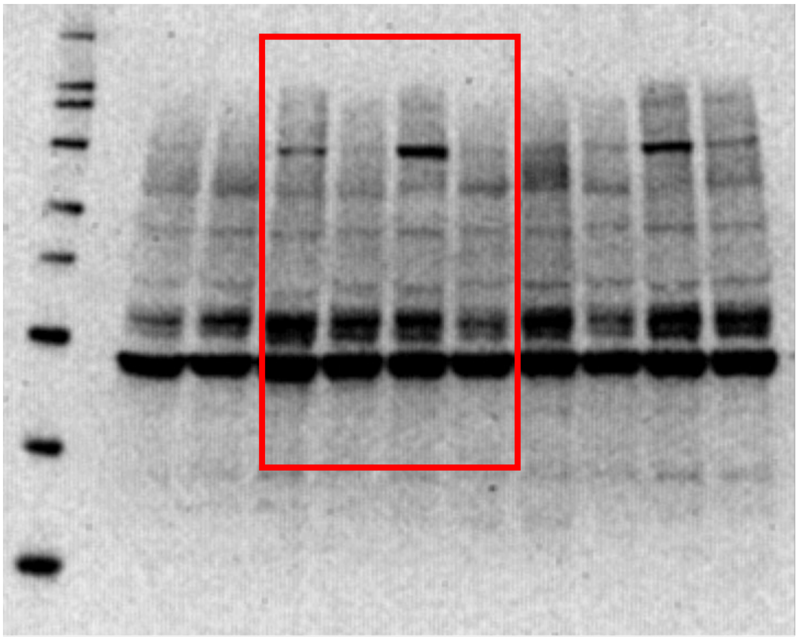

C=control
M=MDD

FIG 4B Versican western blots

RAT

C S C S C* S* C* S* C S C S

C=control
S=CUS

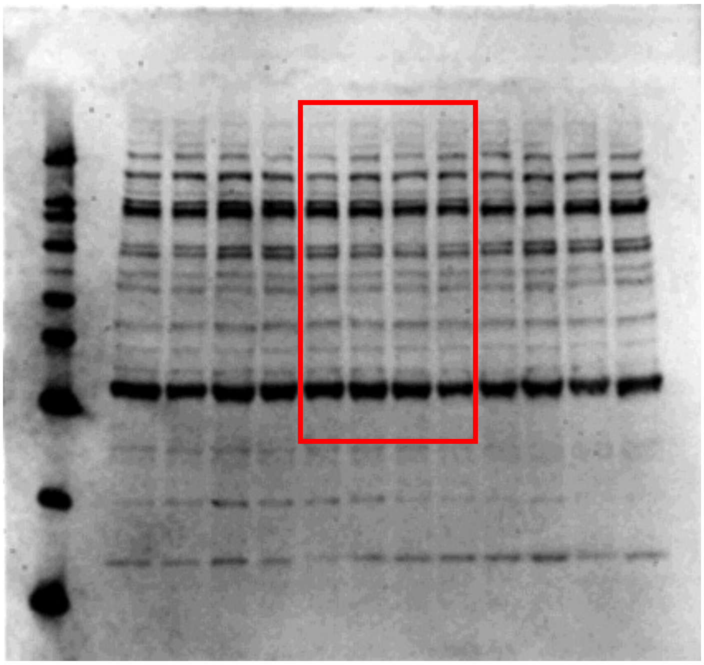

HUMAN

C* M* C* M* C M C M C M

C=control
M=MDD

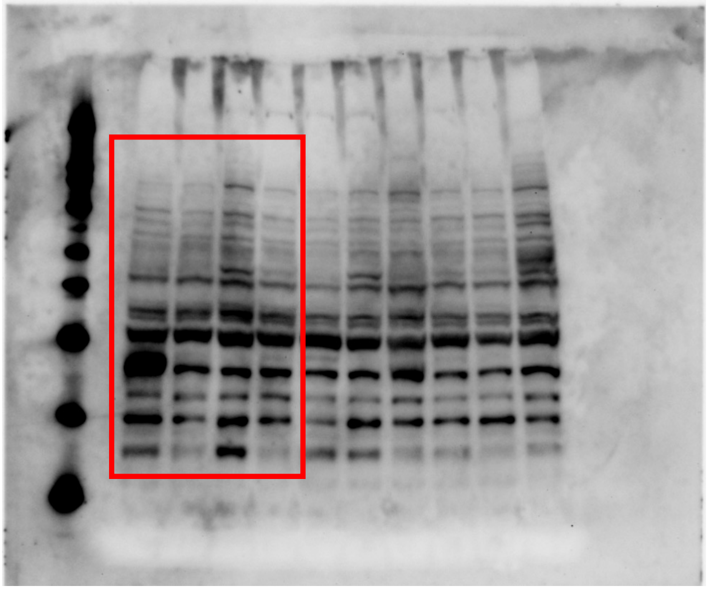

FIG. 5B Brevican western blots

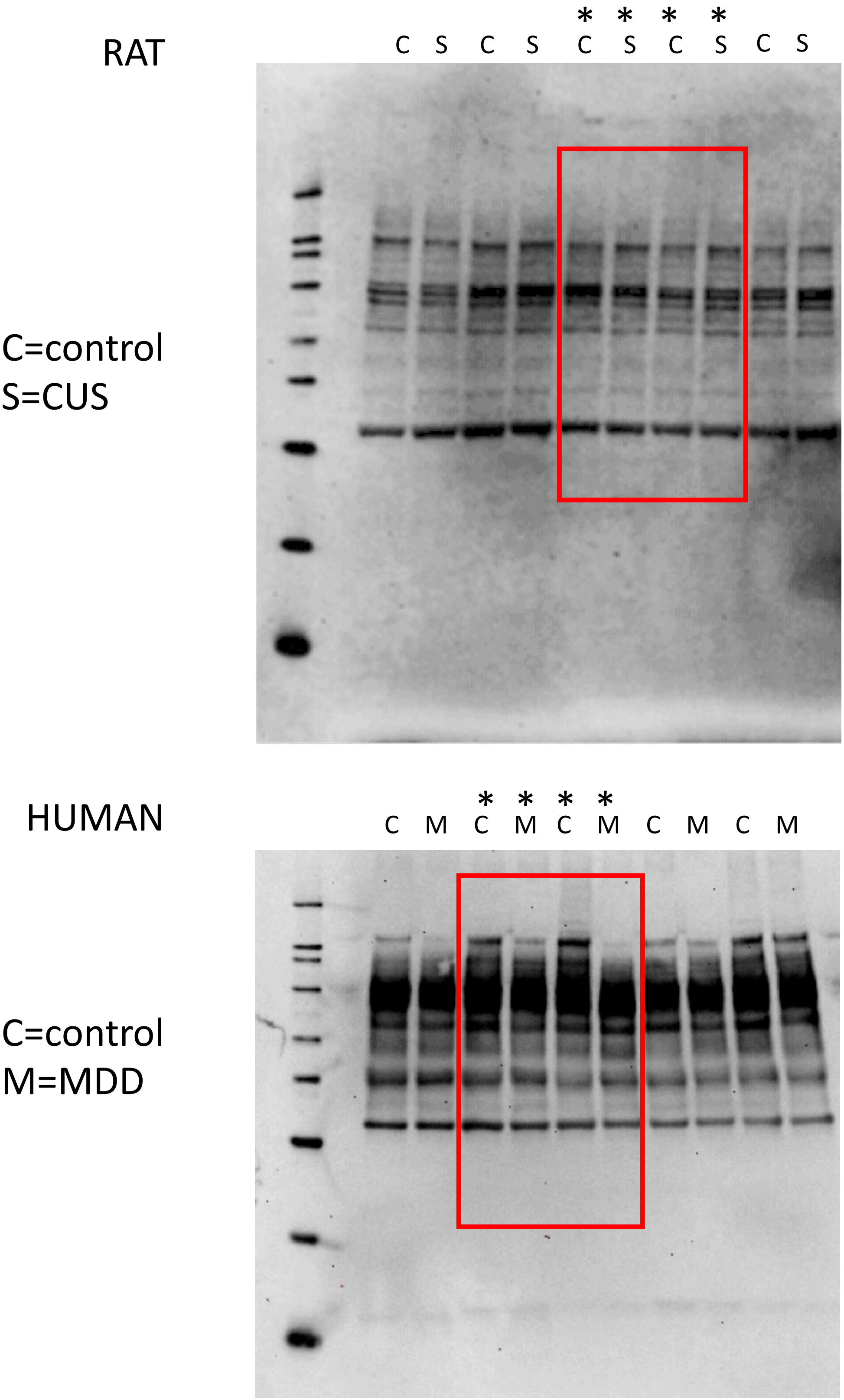

FIG. 6B

BRAL-1 western blots

RAT

* * * *
C S C S C S C S C S C S

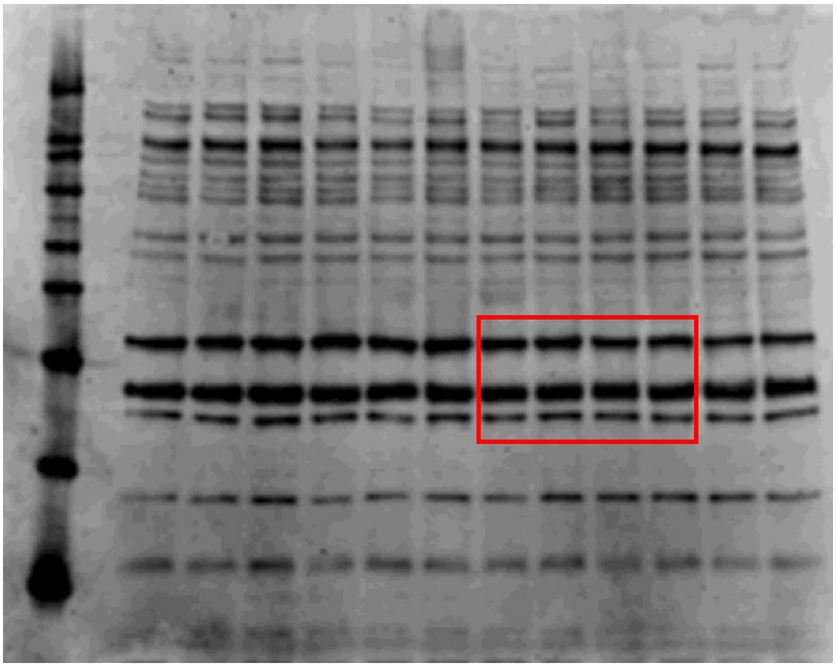

C=control
S=CUS

HUMAN

* * * *
C M C M C M C M C M

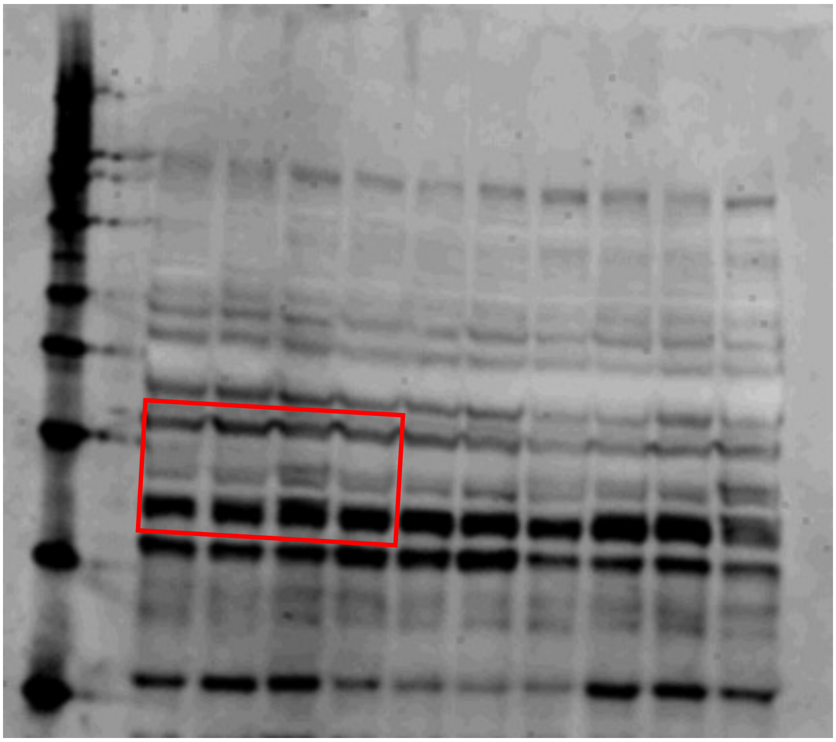

C=control
M=MDD

FIG. 7B Tenascin R western blots

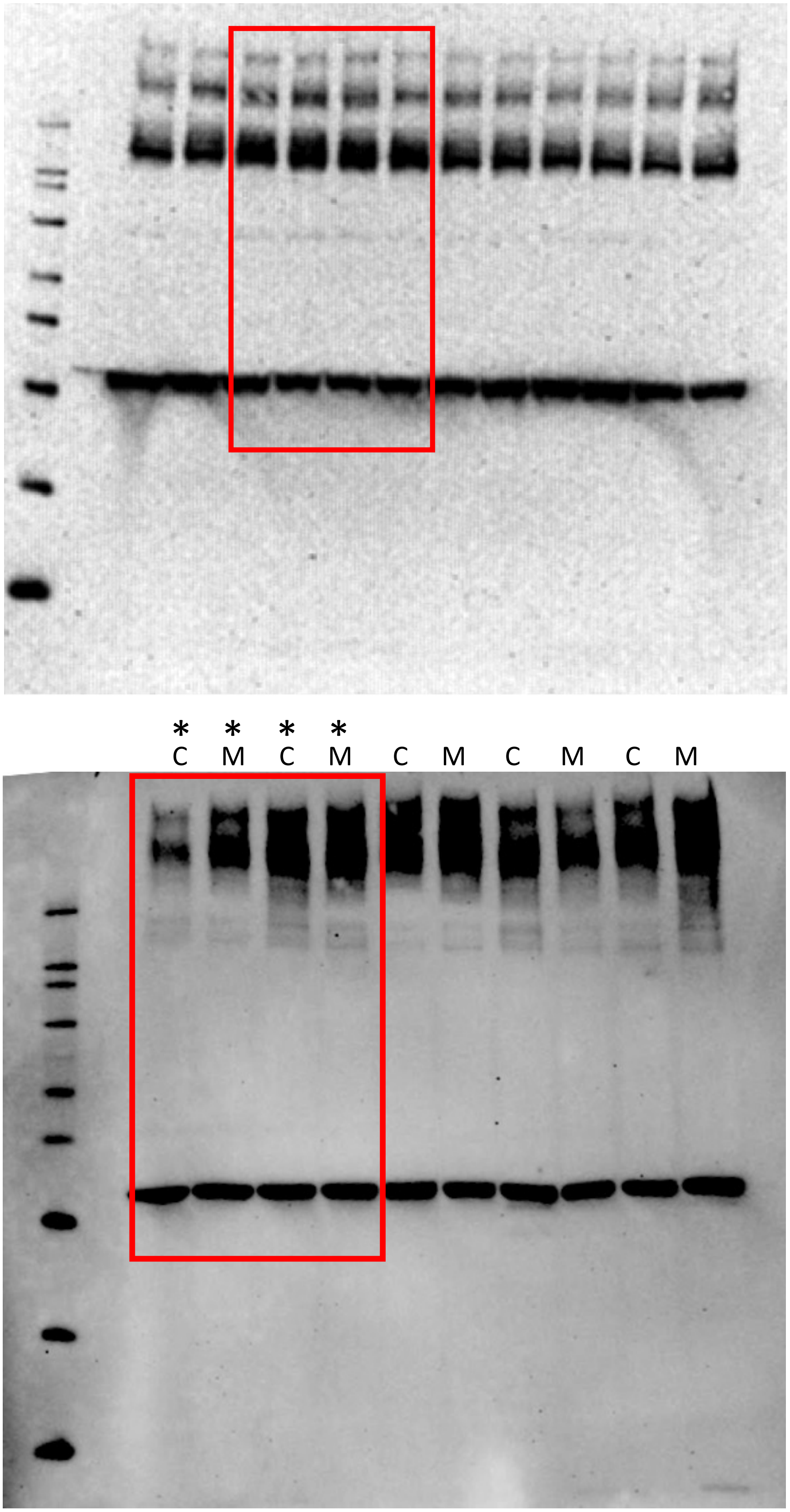

FIG. 8B Neurofascin western blots

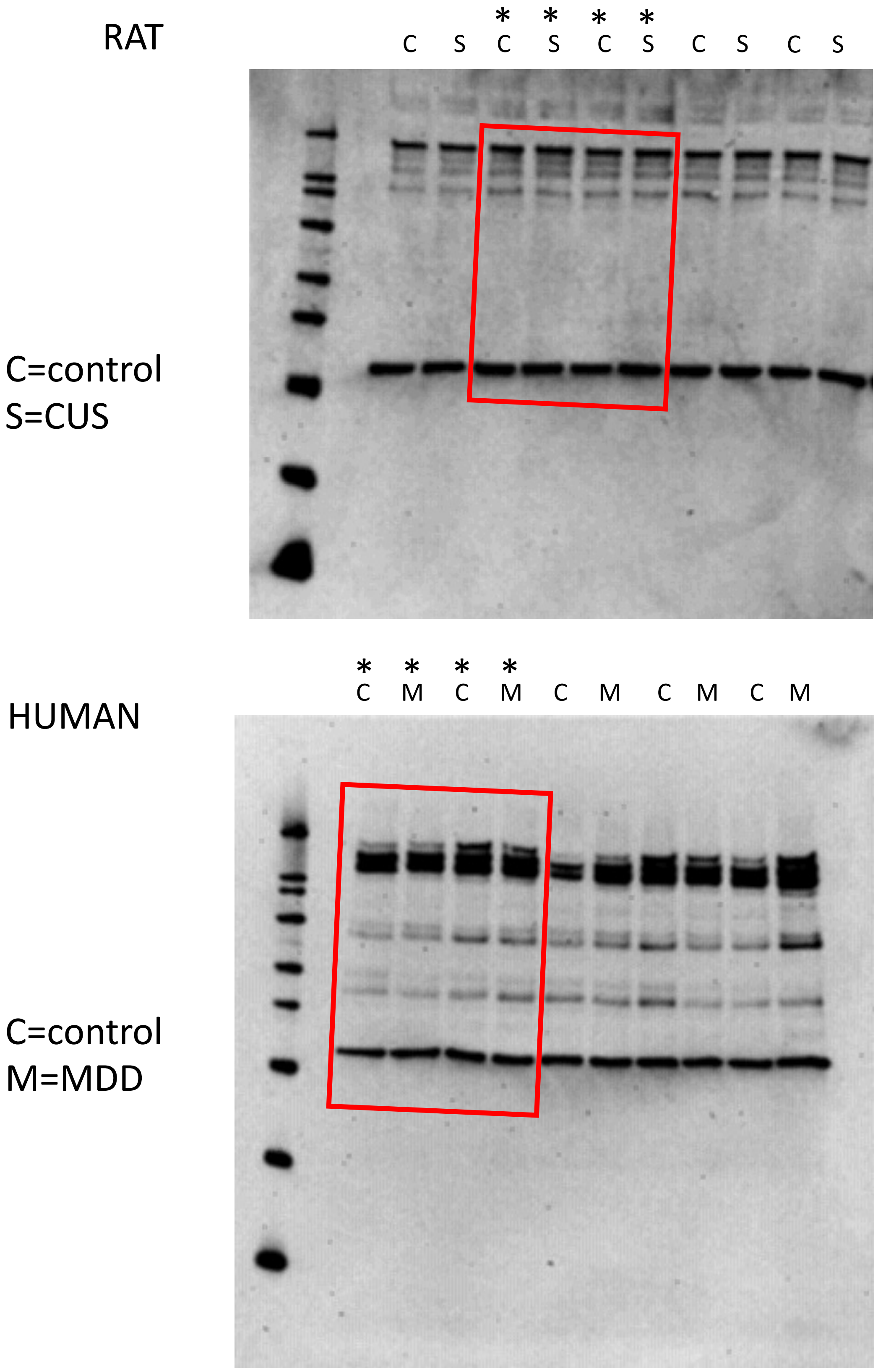

Supplement: Supplementary file 1 — Supplementary Information. [file 41598_2023_43627_MOESM1_ESM.pdf]
